# Dr. James McClintock

**Published:** 1857-08

**Authors:** 


*Dr. James McClintock.*—At a recent meeting of the Board of Guardians, Dr. James McClintock was elected Chief Resident Physician, to the Philadelphia Hospital, Blockly, which appointment caused the resignation of the whole medical corps, with one exception, viz., Dr. A. H. Graham. The Medical and Surgical Reporter says, "In the election of McClintock, the Board has descended into the dirty mire and filth of politics, jeopardizing the interest of an important, medical charity, by offering a deliberate insult, to the respectable medical practitioners, not only of Philadelphia but of the whole country.

